# Peer Review—The Newcomers' Perspective

**DOI:** 10.1371/journal.pbio.0030326

**Published:** 2005-09-13

**Authors:** Gaell Mainguy, Mohammad R Motamedi, Daniel Mietchen

## Abstract

The World Academy of Young Scientists argue that double blind peer-review will generate a better perception of fairness and equality in global scientific funding and publishing

Created under the auspices of the United Nations Educational, Scientific, and Cultural Organization (UNESCO) as an offspring of the “International Forum of Young Scientists,” the World Academy of Young Scientists (WAYS) was officially launched in November 2003 at the World Science Forum in Budapest, Hungary. Our organization represents a permanent global platform for young researchers, and presently gathers some 2,000 members in all disciplines from about 100 countries. WAYS benefits from the support of a number of distinguished senior scientists, including several Nobel laureates. Our objectives are to make science more attractive, comprehensible, and accessible, and to support career development opportunities for young scientists from around the world. WAYS encourages interdisciplinary collaboration and networking among scientists, irrespective of their age or institutional affiliations. We provide a global forum to communicate the opinions, concerns, and questions of young scientists to decision-makers in science policy.

At our first general assembly in December 2004 in Marrakech, Morocco, peer-review procedures in scientific publication and research funding were debated intensely. Even though peer review is universally accepted as an essential element of research, considerable debate persists on how to implement it. The vast majority of our members, especially from developing countries, were concerned about the apparent unfairness of the current procedure, a perception that is prone to generate frustration, fear of discrimination, and distrust. We reached a consensus that slight modifications to the current review process would help in getting more objective reviews based on the quality of the research rather than the age, affiliation, gender, or pedigree of the authors.

Single-blind peer review (SBPR), in which the reviewer knows the identity of the author but not vice versa, is the currently accepted practice. Because SBPR can be vulnerable to sexism and nepotism [1], its ethical foundations have come under criticism; the method is frequently recognized to be biased against new ideas, women, young scientists, career changers, and scholars from less prestigious universities and/or from developing countries (see [2] and references therein). Generally, two policies have been proposed to eliminate bias from the peer-review process: open peer review and double-blind peer review (DBPR).

We believe that current peer-review process, even though functional, can be, and should be, improved.

In open peer review, the identities of both authors and reviewers are revealed, affording the authors the ability to identify the reviewers' comments to a person. Even though this might be an equitable strategy to prevent unfair rejections, this process has no safeguard against unfair acceptance of papers—reviewers, and especially newcomers, may feel pressured into accepting a mediocre paper from a more established lab in fear of future reprisals.

DBPR, in which both the reviewers and the authors remain anonymous to each other, is thought to disentangle the peer-review process from non-scientific factors, thereby presenting an appealing alternative. The a priori case for masking and blinding is strong, and several studies have suggested that articles published in DBPR journals were cited significantly more often than articles published in non-DBPR journals [3,4]. However, other studies have been less convincing; critics of DBPR argue that it is difficult to hide the identity of the institution, laboratory, and/or authors of a paper from the reviewers, especially in smaller specializations. For instance, in a DBPR policy trial, despite explicit instructions to authors, 34% of prospectively evaluated manuscripts contained hints to unblind the authors, and editors correctly identified the authors or institutions of 25% of the manuscripts [5]. The disconnection between principle and practice is evident, and so far, few journals, and even fewer in biomedical sciences, have implemented DBPR policies. The reasons appear to be partly historical, as journals are used to SBPR, and partly intellectual, as the benefits of DBPR still remain controversial [6].

Maintenance of trust within the international scientific community is crucial, not only for future scientific development, but also to continue the dialogue of civilizations. We believe that the current peer-review process, even though functional, can be, and should be, improved to bolster a more even playing field for all scientists. In biomedical sciences, the effectiveness of DBPR is hotly debated. However—using data from computer science, philosophy, or economics, which have adopted and have been using DBPR for some time—the inescapable conclusion is that DBPR performs at least as well as the traditional peer-review process. We propose here that DBPR is a better system because, in addition to being a reasonably fair process, it also bears symbolic power that will go a long way to quell fears and frustrations, thereby generating a better perception of fairness and equality in global scientific funding and publishing. This will, in turn, help to keep research more accessible for future generations.

## Abbreviations

DBPR: double-blind peer review
SBPR: single-blind peer review
WAYS: World Academy of Young Scientists
